# Honokiol Ameliorates High-Fat-Diet-Induced Obesity of Different Sexes of Mice by Modulating the Composition of the Gut Microbiota

**DOI:** 10.3389/fimmu.2019.02800

**Published:** 2019-12-11

**Authors:** Yanan Ding, Zehe Song, Hao Li, Ling Chang, Tingli Pan, Xueling Gu, Xi He, Zhiyong Fan

**Affiliations:** ^1^College of Animal Science and Technology, Hunan Agricultural University, Changsha, China; ^2^Hunan Co-Innovation Center of Animal Production Safety, Hunan Agricultural University, Changsha, China

**Keywords:** honokiol, gut microbiota, metabolites, mice, health

## Abstract

**Background:** Accumulating data support the fact that the gut microbiota plays an important role in the progression of obesity and its related metabolic disease. Sex-related differences are an important consideration in the study of gut microbiota. Polyphenols can regulate gut microbiota, thereby improving obesity and its associated complications. There have been no studies conducted on the ability of honokiol (HON, an extract from Chinese herbal medicine) to regulate gut microbiota. The aim of this study was to examine whether HON supplementation would improve obesity by regulating the gut microbiota and its related metabolite levels, and whether there were sex-based differences in high-fat diet-induced obese mice.

**Methods:** C57BL/6 mice (*n* = 120) were fed a normal chow diet (ND group), high-fat diet (HFD group), or HFD plus HON at 200, 400, and 800 mg/kg BW for 8 weeks. Body weight, adipose tissue weight, adipocyte diameter, insulin resistance, blood lipid and serum inflammatory cytokines, gut microbiota, and its metabolite were examined at the end of the experiment.

**Results:** The HON supplementation reduced body weight, adipose tissue weight, adipocyte diameter, insulin resistance, blood lipid, and serum inflammatory cytokine levels in HFD-fed mice, and this effect was significant in the high-dose group. In addition, HON not only reversed gut disorders in HFD-fed mice, such as by enhanced the abundance of *Akkermansia* and short-chain fatty acids (SCFAs) producing *Bacteroides* and reduced *Oscillospira*, but also improved the SCFAs and endotoxin (LPS) levels, although there were sex-based differences. The correlation between several specific genera and obesity-related indexes was revealed through Spearman's correlation analysis. Moreover, HON may have dose-dependent effects on regulating gut microbiota to alleviate obesity.

**Conclusions:** These findings suggest that HON can prevent diet-induced obesity and its associated diseases by regulating the gut microbiota and improving microbial metabolite levels. Moreover, our findings indicate that sex may be an important factor affecting HON activity.

## Introduction

Obesity and related metabolic diseases, which are caused by a high-fat diet (HFD) and sedentary lifestyle, have been increasing with the improvements in standard of living and the changing lifestyle and have become an urgent public health problem (1). The underlying cause of obesity includes a higher energy storage than consumption due to genetic or environmental factors, as well as an excessive proliferation and differentiation of the body's adipocytes, which ultimately results in weight gain (2). The gut microbiota is made up of trillions of bacteria (3, 4) affecting the nutrient digestion and energy metabolism of the host by regulating nutrient uptake and energy and fat storage. It, therefore, plays an important role in the development of obesity and related metabolic diseases (5, 6). Studies have found that obese people have a less rich microbiota than lean people (7). High-energy and HFD-induced weight gain and fat accumulation were inhibited in germ-free (GF) mice (8, 9). The obese phenotype was also transferred when the microbiota of obese mice was transplanted into GF mice (9). Antibiotic therapy in obese mice reduced obesity and improved glucose metabolism (10). In addition, it was shown that a decrease in *Akkermansia* and *Bacteroides* abundance and an increase in *Oscillospira* abundance are closely related to the occurrence and development of obesity (11–13). These results indicate that microbiota plays an important role in the development of obesity. Moreover, obesity would lead to an increase in serum LPS concentration (14), which is the main component of the outer membrane of Gram-negative bacteria (15). Lipopolysaccharide could lead to metabolic inflammation and insulin resistance (14). Studies have demonstrated that *Akkermansia* can improve the body's inflammatory response and protect the intestinal barrier (11), *Oscillospira* is closely related to the body's inflammatory response and intestinal permeability (13), and *Bacteroides* can promote health by producing short-chain fatty acids (SCFAs) to provide energy for intestinal epithelial cells and inhibit LPS-induced inflammation (12, 16). These results suggest that regulating gut microbiota may be an important measure to prevent obesity and obesity-related metabolic syndrome.

There is increasing evidence that plant functional components, especially polyphenols, can regulate gut microbiota, thereby improving obesity and its associated complications (17, 18). For example, the addition of apple proanthocyanidins significantly increased the ratio of *Bacteroidetes/Firmicutes*, and the abundance of *Akkermansia*, alleviated the weight gain, inflammation, and intestinal permeability, and improved lipid metabolism induced by a HFD (17). Supplementing a basic diet with resveratrol changed the gut microbiota in mice. When this gut microbiota was transplanted into HFD-fed mice, the recipients had less weight gain, increased insulin sensitivity, alleviated inflammation, and improved lipid metabolism and intestinal barrier function (18). In addition, gut microbiota could play a beneficial role in the host by producing SCFAs, such as butyrate, which provide energy for intestinal epithelial cells and inhibit inflammation induced by LPS, promoting health (12, 16). These results suggest that polyphenols can improve obesity and related complications by regulating gut microbiota and metabolites.

Honokiol (HON) is a naturally occurring, pleiotropic lignan that was confirmed as one of the main biological active components of *Magnolia officinalis*, a traditional Chinese medicine (19). HON has been shown to have broad-spectrum antibacterial (20), anti-tumor (21), neuro-modulating (19), and anti-oxidation activities (22) by modern pharmacological tests, and has now become more widely studied due to its pleiotropic effects. In previous reports, supplementation of HON and magnolol ameliorated body fat accumulation, insulin resistance, and adipose inflammation in HFD-fed mice (23). However, only 5–10% of HON are absorbed in the small intestine as dietary polyphenol. The rest are transported to the posterior intestine for microbial degradation and utilization, and shape the structure of gut microbiota (24). Therefore, it cannot be ignored that the role of gut microbiota in the process of HON acting on the host. In addition, studies have confirmed that sex-related differences are an important consideration in the study of gut microbiota. For example, women had fewer metabolic disorders caused by diet (25, 26), and inflammatory bowel disease caused by microbiota changes in the gut was more common in women than in men (27, 28). However, whether there are sex-related in the regulation of high-fat-induced gut microbiota, improving obesity and related complications by HON, has not been studied.

Therefore, the aim of this study was to investigate whether HON can improve obesity and its related complications by regulating gut microbiota, and whether this effect was associated with sex differences.

## Materials and Methods

### Materials

HON samples (purity > 99.9%) were purchased from Victory Biological Technology Co., Ltd. (Sichuan, China).

### Animal and Experimental Design

After approval by the Committee on the Ethics of Animal Experiments of Hunan Agriculture University, 120 C57BL/6 mice (SPF, 6 weeks old, 60 male and 60 female) were purchased from the Hunan SJA Laboratory Animal Co., Ltd (Hunan, China). All mice were housed 2/cage at 23 ± 2°C with 50 ± 5% relative humidity and with a 12-h light–dark cycle. After 1 week of acclimation, the mice were randomly divided into five groups randomly (24 mice per group, half male and half female), and fed a normal chow diet (ND group, D12450J, 10% kcal from fat, 3.85 kcal/g, Research Diets, Inc., USA), high-fat diet (HFD group, D12492, 60% kcal from fat, 5.24 kcal/g, Research Diets, Inc., USA), and HFD with HON at 200 mg/kg BW (H200 group), 400 mg/kg BW (H400 group), and 800 mg/kg BW (H800 group) for 8 weeks. Body weight was measured weekly, and food intake was recorded daily. After 8 weeks of experimental treatment, blood samples were collected from the orbital plexus and the mice were sacrificed. The adipose tissues were removed, rinsed with a physiological saline solution, weighed, and stored at −80°C until analyzed. Meanwhile, cecal contents were collected in Eppendorf tubes and immediately stored at −80°C for subsequent analysis. The animal experimental protocols were conducted in accordance with the Institutional Animal Care and Use Committee of Hunan Agriculture University.

### Insulin Resistance Assessment

The blood insulin of fasted mice (overnight) was measured using a commercial ELISA kit (Wuhan Cusabio biotech Co., Ltd., China), according to the manufacturer's recommendations. Blood glucose was determined with an automatic biochemical analyzer (Shenzhen Mindray Bio-Medical Electronics Co., Ltd., China). The homeostasis model assessment-insulin resistance (HOMA-IR) was calculated using the following formula: HOMA-IR = fasting blood glucose (mmol/L) × fasting insulin (mU/L)/22.5.

### Biochemical Analysis and Cytokine Measurements

Serum triglyceride (TG), total cholesterol (TC), low-density lipoprotein cholesterol (LDL-C), and high-density lipoprotein cholesterol (HDL-C) were determined by an automatic biochemical analyzer (Shenzhen Mindray Bio-Medical Electronics Co., Ltd., China). Free fatty acids (FFA) were determined using a commercial kit (Nanjing Jiancheng Bioengineering Institute, Nanjing, China). Serum LPS-binding protein (LBP), tumor necrosis factor-α (TNF-α), interleukin-6 (IL-6), interleukin-1β (IL-1β), and interferon-γ (IFN-γ) concentrations were then quantified using commercial ELISA kits (Wuhan Cusabio biotech Co., Ltd., China).

### Histological Examination

Epididymal WAT was dissected, washed in saline, and immediately fixed in 4% paraformaldehyde. Fixed tissues were embedded in paraffin and 4-μm sections were prepared and stained with hematoxylin and eosin (H&E) for general morphological observations. Images were acquired at 200 × magnification. The sizes of white adipose tissue was measured using Photoshop CS6 (Adobe Systems Inc., San Jose, CA, USA).

### SCFA Analysis

About 60 g of cecal contents were weighted accurately using a 1/10000 balance, added to 1.5 ml of ultrapure water, and vortexed uniformly for 2 min overnight. The mixtures were then centrifuged at 12,000 *g* for 15 min, and the supernatants and 25% metaphosphoric acid were mixed in a proportion of v:v = 9:1 for SCFA analysis. The SCFA content was determined using an Agilent 7890A at a programmed temperature (Agilent Technologies Inc., USA).

### Gut Microbiota Analysis

Five cecal content samples from each group were selected for microbiota 16S rRNA analysis. Total bacterial genomic DNA samples were extracted using a Fast DNA SPIN extraction kit (MP Biomedicals, Santa Ana, CA, USA), following the manufacturer's instructions. The quantity and quality of the extracted DNAs were measured using a NanoDrop ND-1000 spectrophotometer (Thermo Fisher Scientific, Waltham, MA, USA) and agarose gel electrophoresis, respectively. The V3–V4 hypervariable region of the bacterial 16S rRNA gene was amplified using universal primers (338F and 806R). Sequencing was performed on the Illumina HiSeq platform using HiSeq2500 PE250 (Illumina, USA).

The Quantitative Insights Into Microbial Ecology (QIIME, v1.8.0) pipeline was used to process the sequence data. Briefly, raw sequencing reads with exact matches to the barcodes were assigned to their respective samples and identified as valid sequences. Low-quality sequences were filtered according to the criteria of Chen and Jiang (29). After chimera detection, the remaining high-quality sequences were clustered into operational taxonomic units (OTUs) at 97% sequence identity by UCLUST (30). The abundance of each OTU in each sample and the taxonomy of these OTUs are represented by an OTU table. Data analyses were mainly performed using QIIME and R packages (v3.2.0). OTU-level alpha diversity indices, such as the Chao1 richness estimator, ACE metric (Abundance-based Coverage Estimator), Shannon diversity index, and Simpson index, were calculated using the OTU table in QIIME. Beta diversity analysis was used to investigate the structural variation of microbial communities across samples using UniFrac distance metrics (31) and visualized via principal coordinate analysis (PCoA), nonmetric multidimensional scaling (NMDS), and the unweighted pair-group method with arithmetic means (UPGMA) hierarchical clustering (32). Venn diagrams were generated to visualize the shared and unique OTUs among samples or groups using the R package Venn Diagram, based on the occurrence of OTUs across samples/groups regardless of their relative abundance (33). A heatmap was created on the basis of the top 50 dominant genera using R packages (V3.5.2). The Spearman's rho nonparametric correlations between the gut microbiota and obesity-related indexes were determined using R packages (V3.5.2).

### Statistical Analysis

Data are expressed as the mean ± SEM (the standard error of the mean). A two-sided unpaired Student's *t*-test with Benjamini–Hochberg correction was used to compare the two groups. The differences among the four groups were analyzed using one-way analysis of variance (ANOVA) followed by Duncan's test. Significance was set at *P* < 0.05.

## Results

### HON Suppresses HFD-Induced Increases in Body Weight of Mice

**Figure 1:**
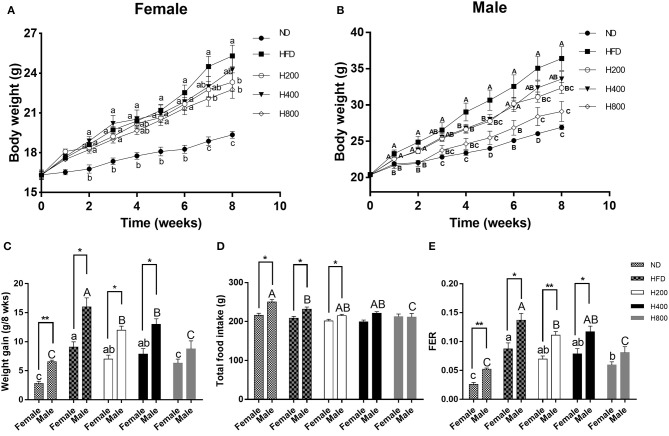
Effect of HON on body weight and food intake in HFD-fed mice. Mice were fed with a normal chow diet (ND), a high-fat diet (HFD), HFD plus HON at 200 (H200), 400 (H400), and 800 (H800) mg/kg BW for 8 weeks. Changes in the **(A,B)** body weight; **(C)** body weight gain; **(D)** total food intake; and **(E)** FER. Mean ± SE (*n* = 12). HON, honokiol. Statistically significance results between female and male treatment groups were expressed by lowercase letters (a, b, c) and uppercase letters (A, B, C) based on ANOVA with Duncan's range tests. Mean values with different letters indicate statistically significance results (*P* < 0.05). Statistically significant results in different sexes group are marked by ^*^P < 0.05 and ^**^P < 0.01.

In female and male mice, the body weight and weight gain of HFD-fed mice were significantly higher than those of the ND-fed and HFD + 800 mg/kg HON-fed mice at the end of study (*P* < 0.05, Figures 1A–C). The HFD + 200 mg/kg HON diet had similar effects on male mice (*P* < 0.05, Figures 1A–C). In addition, while the weights of the female mice in the H800 group significantly decreased compared to those of female mice in the HFD group by 7 weeks, the difference in the weights of the male mice was seen 4 weeks earlier (*P* < 0.05, Figures 1A,B). Total food intake did not vary significantly among the HFD-fed groups of female mice and male mice except for the H800 group (Figure 1D), where a significant decrease of food efficiency ratio (FER) was observed for the H800 group of female mice and H800 and H200 groups of male mice compared with the HFD group (*P* < 0.05, Figure 1E), suggesting that the effects of HON on body weight did not result from the reduction of food intake but from the FER (weight gain divided by food consumption weight). These results imply that HON reduced weight gain in HFD-fed mice and that there were sex-based differences in the body weight, weight gain, and FER (*P* < 0.05, Figures 1C–E); Moreover, the food intake of HFD + 800 mg/kg HON male mice was notably lower than that of the female mice (*P* < 0.05, Figure 1D).

### HON Prevents Increases in Adipose Tissue Mediated by a HFD in Mice

**Figure 2:**
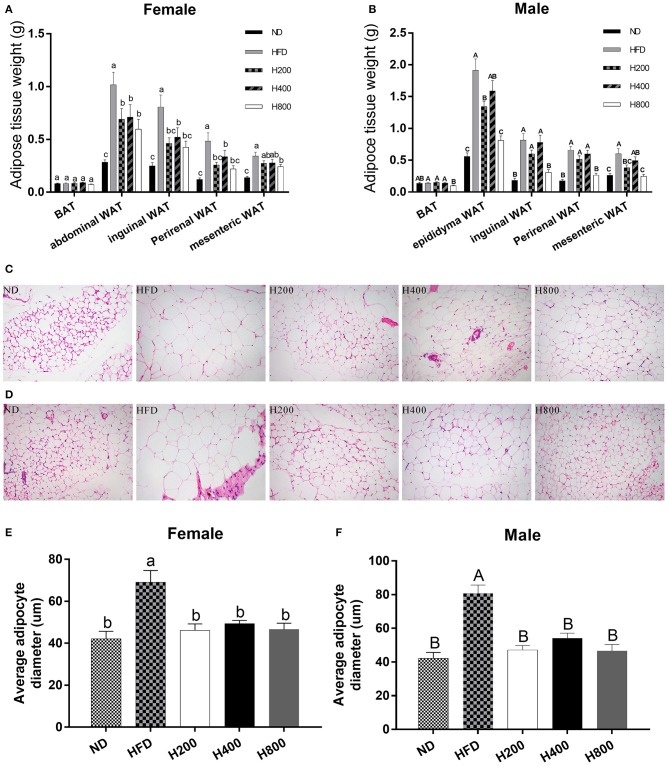
Effect of HON on adipose mass gain. **(A)** Changes in fat pad weights of abdominal white adipose tissue (WAT), **(B)** epididymal WAT, **(A,B)** inguinal WAT, **(A,B)** perirenal WAT, **(A,B)** mesenteric WAT and **(A,B)** brown adipose tissue (BAT). Mean ± SE (*n* = 12). Inguinal WAT; Average adipocyte diameter of inguinal WAT ×200 magnification **(C–F)**. ND, normal chow diet; HFD, high-fat diet; HON, honokiol. H200, H400, and H800 correspond to honokiol supplemented with 200, 400, and 800 mg/kg in high fat diet, respectively. Statistically significance results between female and male treatment groups were expressed by lowercase letters (a, b, c) and uppercase letters (A, B, C) based on ANOVA with Duncan's range tests. Mean values with different letters indicate statistically significance results (*P* < 0.05).

Since HON decreases the body weight of HFD-fed mice, we investigated whether the decreased body weight gain was due to a reduction in adipose tissue mass by weighing the fat pads. As shown in Figures 2A,B, the HFD significantly increased weights of the abdominal WAT, epididymal WAT inguinal WAT, perirenal WAT, and mesenteric WAT fat pads over those of the ND-fed group. HON supplementation decreased the HFD-induced gains in WAT weights, especially at high doses (*P* < 0.05, Figures 2A,B). Histological sections from the inguinal WAT of HFD group showed that adipocyte diameter was greater in the HFD than the ND group, while adipocyte size was clearly smaller in the H200, H400, and H800 groups than in the HFD group (*P* < 0.05, Figures 2C–F). Adipose mass was correlated with body weight in the mice fed HON, suggesting that the HON-induced decrease in body weight increase could be attributed to a reduction in adipose tissue weights. Results showed that HON significantly decreased adipocyte diameter in inguinal WAT compared to the HFD group (*P* < 0.05, Figures 2C–F), suggesting that HON may inhibit adipogenesis and hyperplasia of inguinal WAT.

### HON Ameliorates Serum Lipid Profile and Attenuates Systematic Inflammation in HFD-Fed Mice

**Figure 3:**
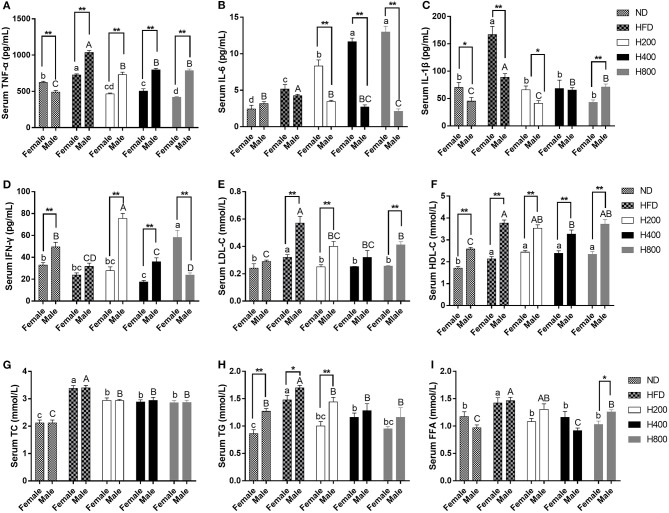
Effects of HON ameliorates serum lipid profile and attenuates systematic inflammation in HFD-fed Mice. Serum levels of TNF-α **(A)**, IL-6 **(B)**, IL-1β **(C)**, IFN-γ **(D)**, LDL-C **(E)**, HDL-C **(F)**, TC **(G)**, TG **(H)**, FFA **(I)**. ND, nomal chow diet; HFD, high fat diet; HON, honokiol. H200, H400, and H800 correspond to honokiol supplemented with 200, 400, and 800 mg/kg in high fat diet, respectively. Statistically significance results between female and male treatment groups were expressed by lowercase letters (a, b, c, d) and uppercase letters (A, B, C, D) based on ANOVA with Duncan's range tests. Mean values with different letters indicate statistically significance results (*P* < 0.05). Statistically significant results in different sexes group are marked by ^*^P < 0.05 and ^**^P < 0.01.

Obesity is accompanied by increased serum lipid levels and inflammatory cytokine levels (34). As shown in Figure 3, compared to the ND-fed group, mice in the HFD-fed group had significantly increased serum levels of TG, TC, HDL-C, LDL-C, FFA, TNF-α, IL-6, and IL-1β, and decreased IFN-γ serum levels. HON supplementation remarkably reduced serum lipid levels (TG, TC, FFA, and LDL-C) and systematic inflammation (TNF-α and IL-1β) compared to those in the HFD group (*P* < 0.05). HFD plus HON at 400 and 800 mg/kg significantly increased serum levels of IL-6 and decreased FFA concentration (*P* < 0.05, Figure 3). In addition, male mice had lower serum levels of IL-6 and IL-1β than female mice and there was no significant difference between TC and FFA.

### HON Reduces Insulin Resistance in HFD-Fed Mice

**Figure 4:**
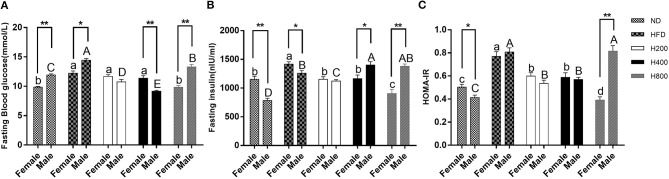
Preventive effects of HON on the development of insulin resistance in HFD-fed mice. Fasting blood glucose **(A)**, fasting insulin **(B)**, and HOMA-IR **(C)**, calculated according to the formula: fasting blood glucose (mmol/L) ×fasting insulin (mIU/L)/22.5 are shown. ND, normal diet; HFD, high-fat diet; HON, honokiol. H200, H400, and H800 correspond to honokiol supplemented with 200, 400, and 800 mg/kg in high fat diet, respectively. Statistically significance results between female and male treatment groups were expressed by lowercase letters (a, b, c, d) and uppercase letters (A, B, C, D) based on ANOVA with Duncan's range tests. Mean values with different letters indicate statistically significance results (*P* < 0.05). Statistically significant results in different sexes group are marked by ^*^P < 0.05 and ^**^P < 0.01.

Studies indicated that diet-induced obese mice would have significantly increased fasting glucose and an elevated HOMA-IR value (34). In the present study, all the HON treatments, except for the high dose of HON on male mice, resulted in a significant reduction in fasting glucose and insulin levels along with a decrease in HOMA-IR in HFD-fed mice (*P* < 0.05, Figures 4A–C), suggesting that HON can improve insulin resistance. In addition, there were sex-related in fasting glucose and HOMA-IR.

### Effects of HON Treatment on the Gut Microbiota Composition in Diet-Induced Obese Mice

**Figure 5:**
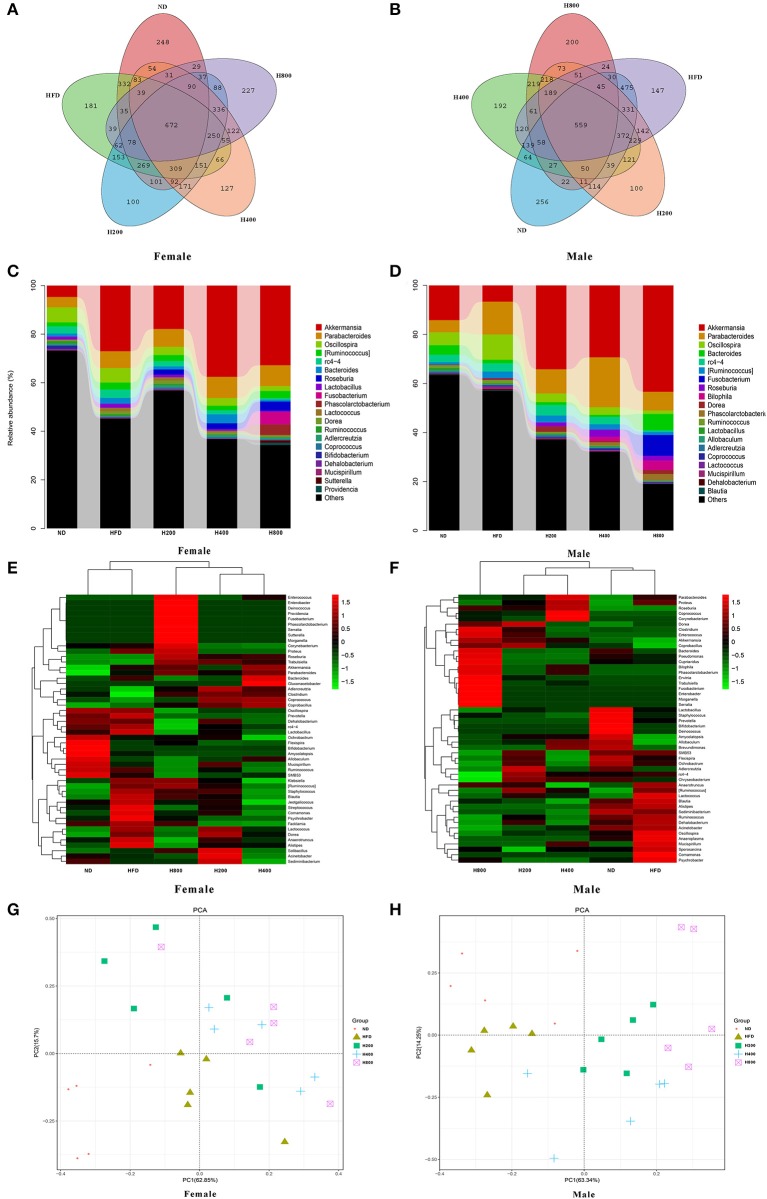
HON alter the composition of the gut microbiota in HFD-fed mice. Venn diagrams showing the unique and shared OTUs in the gut microbiota of the groups **(A,B)**. Community taxonomic composition and abundance distribution map at genus level **(C,D)**. Hierarchically clustered heat map analysis of the top 50 most abundant of gut microbes at genus level **(G,H)**. PCA clustering analysis **(E,F)**. H200, H400, and H800 correspond to honokiol supplemented with 200, 400, and 800 mg/kg in high fat diet, respectively.

Increasing evidence shows that the gut microbiota plays an important role in the development of obesity and obesity-related complications (6, 35). Therefore, after 8 weeks of feeding, we sequenced cecal content samples to elucidate the effects of ND, HFD, and HFD supplemented with HON on the gut microbiota structure. In female mice, at all detected, 672 OTUs were detected in all groups (Figure 5A). There were 248, 181, 100, 127, and 227 unique OTUs in the ND, HFD, H200, H400, and H800 groups, respectively. There was no significant difference in the value of ACE, Chao1, Shannon, and Simpson between the ND, HFD, H200, and H400 groups (Table S1). However, significant differences in ACE and Chao1 were found between mice in the HFD and H800 groups (Table S1). In male mice, 559 OTUs were presented in all groups (Figure 5B). There were 256, 147, 100, 192, and 200 unique OTUs in the ND, HFD, H200, H400, and H800 groups, respectively. Significant differences were also found in the ACE, Chao1, Shannon, and Simpson measures of the HFD and H800 groups (Table S3). PCA revealed a significant separation on the microbiota of the groups (Figure 5G). Collectively, these results indicated that HON modulated the gut microbiota of HFD-fed mice. Moreover, the HFD-fed group had significantly reduced *Muribaculaceae* and *Bifidobacterium*, and significantly increased *Oscillospira* and *Lactococcus* (*P* < 0.05, Figures 5D,E and Table S4), and decreased *Akkermansia* but not significant compared to the ND group (Figures 5D,E and Table S4). The changes in *Akkermansia* and *Oscillospira* were opposite to those in the female mice (Figures 5C–F and Table S2). Moreover, compared to the HFD-fed group, the abundances of *Akkermansia* in all HON treatment, as well as *Bacteroides* (the SCFA-producing microbiota), *Bilophila, Unclassified_Enterobacteriaceae*, and *Fusobacterium* in the H800 group were remarkably enriched. While *Muribaculaceae, Oscillospira*, and *Ruminococcus* in all HON treatments, and *Unclassified_Clostridiales, Unclassified_Ruminococcaceae, rc4-4, Lactococcus*, and *Dehalobacterium* in the H800 group were sharply reduced (*P* < 0.05, Figures 5D,H and Table S4). Notably, *Akkermansia*, which belongs to the phylum *Verrucomicrobia*, was enriched and *Unclassified_Clostridiales*, which belongs to the Phylum Fusobacteria, were reduced by HON supplementation in HFD-fed male mice but not female mice.

### HON Improves Related Gut Metabolites in HFD-Fed Mice

**Figure 6:**
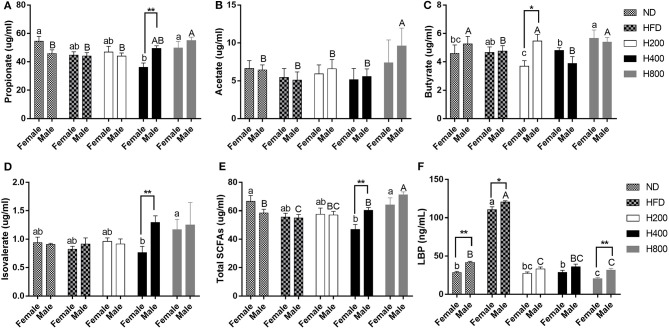
HON improve related gut metabolites in HFD-fed mice. Concentrations of propionate **(A)**, acetate **(B)**, butyrate **(C)**, isovalerate **(D)**, total SCFAs **(E)**, LBP **(F)**. ND, nomal chow diet; HFD, high fat diet; HON, honokiol. H200, H400, and H800 correspond to honokiol supplemented with 200, 400, and 800 mg/kg in high fat diet, respectively. Statistically significance results between female and male treatment groups were expressed by lowercase letters (a, b, c) and uppercase letters (A, B, C) based on ANOVA with Duncan's range tests. Mean values with different letters indicate statistically significance results (*P* < *0.05*). Statistically significant results in different sexes group are marked by ^*^P < 0.05 and ^**^P < 0.01.

Since gut microbiota was modulated by HON supplementation in HFD-fed mice, we next investigated the effect of HON on microbial metabolites, SCFAs, and LBP. The HFD-fed group had a reduced concentration of total SCFAs and increased concentration of LBP in the cecum compared to ND-fed group (*P* < 0.05, Figures 6E,F). HON treatment increased the cecum concentration of SCFAs especially high dose of HON treatment, which markedly increased concentrations of propionate, acetate, butyrate, and total SCFAs, and significantly decreased concentrations of LBP (*P* < 0.05, Figures 6A–C,E,F). Since SCFAs could effectively inhibit the release of LPS endotoxin as shown in Figure 6, HON treatment significantly reduced the concentration of LBP in mice (*P* < 0.05), thereby reducing metabolic endotoxemia in HFD-fed mice, and there were sex-related in LBP (36).

### Correlation of Gut Microbiota With Obesity-Associated Indexes

**Figure 7:**
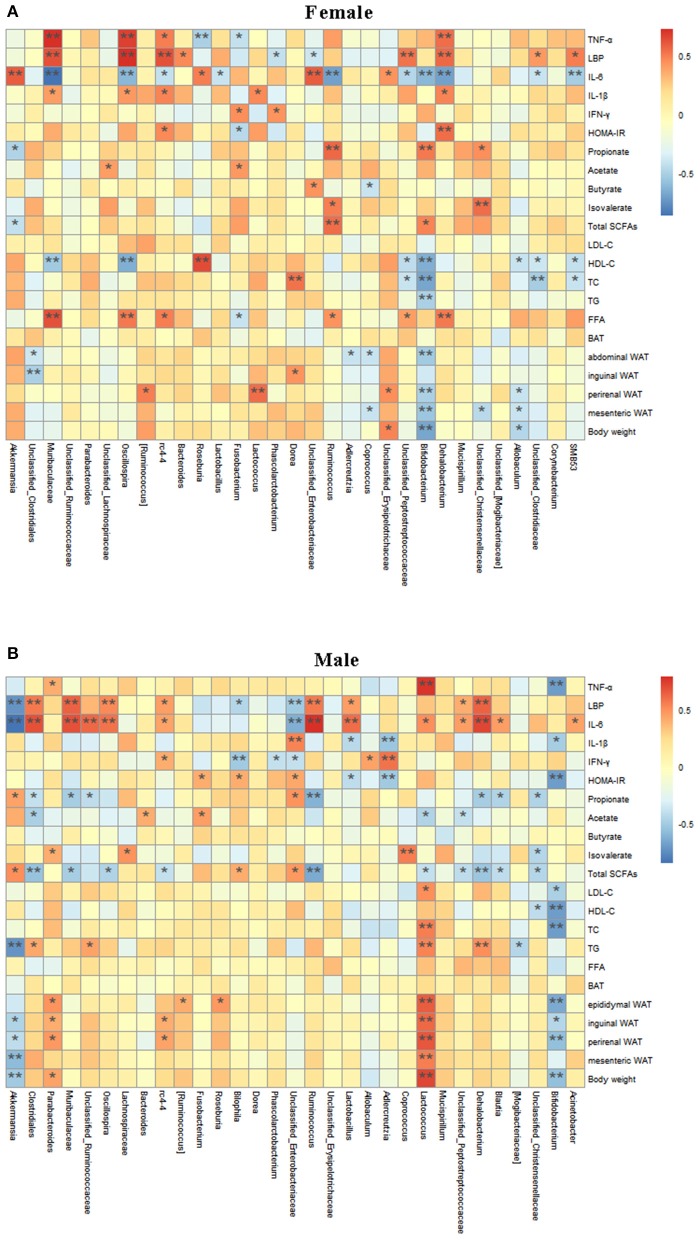
Relationship between intestinal microbiota and Obesity-related Indexes analyzed with a Spelman correlation heatmap. Analysis of correlation between gut microbiota at the genus level and obesity-related indexes are shown in the **(A,B)**. The colors range from blue (negative correlation) to red (positive correlation). ^*^P < 0.05 and ^**^P < 0.01.

Spearman correlation analysis was used to determine the potential correlation between gut microbiota and obesity-related indexes (Figure 7). There was a significant positive correlation between *Dehalobacterium, Oscillospira, rc4-*4, *Muribaculaceae*, and TNF-α, LBP, and IL-1β in female mice (Figure 7A). *Bifidobacterium* was negatively correlated with body weight, mesenteric fat and GLU, TG, TC, and HDL-C. *Akkermansia* was negatively correlated with LBP (Figure 7A). In male mice (Figure 7B), *Ruminococcus, Oscillospira Muribaculaceae, Dehalobacterium, Lactobacillus*, and *rc4-4* showed a significant positive correlation with LBP and IL-6, while *Unclassified_Enterobacteriaceae* were significantly negatively correlated with LBP and IL-6. *Bifidobacterium* showed a significant negative correlation with body weight, perinenal WAT, inguinal WAT, epididymal WAT, TC, HDL-C, LDL-C, HOMA-IR, IL-1β, and TNF-α, while *Lactococcus* showed a significant positive correlation with body weight, mesenteric WAT, perinenal WAT, and epididymal WAT, TC, HOMA-IR, TNF-α, and IL-6. Notably, as a genus that was differentially regulated by the treatments, *Akkermansia* was significantly negatively correlated with body weight, perinenal WAT, inguinal WAT, TG, LBP, and IL-6, and positively correlated with total SCFAs, and propionate. These results suggest that *Akkermansia* and *Bifidobacterium* may play an important role in the prevention of obesity and related complications, while *Lactococcus* may promote the occurrence and development of obesity. *Dehalobacterium, Oscillospira, Muribaculaceae, rc4-4*, and *Ruminococcus* may be closely related to the occurrence of obesity. In addition, there were sex-related in the *Akkermansia* abundance.

## Discussion

Obesity is a common metabolic disease accompanied by numerous health problems (1). As a natural bioactive substance, polyphenols can prevent diet-induced obesity and related complications by regulating the composition of gut microbiota (17, 18). Previous studies have confirmed that HON can improve diet-induced liver lipid metabolism in obese mice (37, 38). Nevertheless, the effects of it on gut microbiota, and its ability to treat obesity and related complications by regulating the composition of gut microbiota have not previously been reported. In this study, we demonstrated that HON can alleviate obesity and its related complications induced by a HFD by altering gut microbiota, and indicated that sex may be a vital factor affecting the improvement of gut microbiota composition by HON.

A HFD can induce obesity and associated complications in mice, such as excessive fat accumulation, dyslipidemia, inflammation, and increased insulin resistance (39). HON supplementation can reverse the occurrence of high-fat-induced obesity and the above related obesity complications in mice. This suggests that HON has a beneficial effect on obesity, and which is achieved not by lowering food intake, but by reducing the FER in mice. Only a small portion of polyphenols can be absorbed by the small intestine, with most decomposed and utilized by microbiota in the hindgut (24). Recently, a rapidly expanding field of research has shown that the gut microbiota is a significant factor in obesity through the regulation of nutritional access, energy distribution and fat storage (5). Moreover, high fat could bring about an imbalance of gut microbiota in obese people (6) and the gut microbiota has been recognized as a crucial environmental factor in the pathogenesis of obesity (3). These results suggest that polyphenols may improve obesity and its related complications by affecting the gut microbiota.

In addition, when gut microbiota detection was performed on animals with specific genetic background, the results were more evident than those considering different sexes, which was also the reason for inconsistent conclusions in relevant studies (40, 41). Therefore, when observing the interaction between the gut microbiota and environmental factors, such as diet, sex as a crucial factor is worth considering (42, 43). The evidence indicates that women were less likely to suffer from diet-related metabolic disorders than men (25, 26).

*Akkermansia* is a genus of Gram-negative anaerobic bacteria belonging to the *Verrucomicrobia*, and its abundance is significantly negatively correlated with obesity. Sustained intervention with a HFD in mice for 8 weeks resulted in a 100-fold reduction in the abundance of *Akkermansia* in the cecum (44). The alteration in *Akkermansia* abundance was correlated with changes in lipid metabolism and the expression of inflammatory response markers (45). *Akkermansia* intervention could improve obesity and related complications such as fat accumulation, insulin resistance, and circulating levels of inflammatory factors induced by a HFD (44). These results indicate that *Akkermansia* play an important role in improving obesity and related complications. In addition, it has been demonstrated that polyphenols can improve obesity and related complications by changing the abundance of *Akkermansia* in the gut (17). In this study, we demonstrated that a HFD resulted in a significant decrease in cecum *Akkermansia* abundance and that supplementation with HON led to a significant increase in *Akkermansia* in male mice. In addition, HON decreased adipose tissue weight and serum inflammatory factor levels, and improved insulin resistance and dyslipidemia in HFD-fed male mice. Correlation analysis also discovered that *Akkermansia* were negatively correlated with body weight, adipose tissue weight of WAT and TG, LBP, and IL-6, which is consistent with previous studies. These results suggest that HON can improve obesity and related complications by increasing the abundance of *Akkermansia* in the gut. It is worth noting that the HFD increased the cecum abundance of *Akkermansia*, and HON supplementation did not have a significant impact on their richness in female mice. This result was contrary to what was seen in male mice, and the correlation analysis of *Akkermansia* with obesity and related indexes was also different in male and female mice, indicating that *Akkermansia* abundance may be associated with sex. The study also confirmed that the abundance of *Akkermansia* was varied in mice of different sexes (43, 46), which was related to the secretion levels of hormone and bile acid (43).

*Bacteroides* is a Gram-negative anaerobic belonging to *Bacteroidetes*, which produce SCFAs. *Bacteroides* abundance was related to the occurrence and development of obesity and a HFD reduce its abundance (12, 47). A Mediterranean diet is associated with more *Bacteroides* than the Western diet (48). Oral *Bacteroides uniformis* significantly reduced body weight, increased the number of adipocytes, and improved glucose tolerance, dyslipidemia, and the immune response in obese mice (49). Besides, the abundance of *Bacteroides* was correlated with sex (50); it decreased with the increasing body mass index (BMI) in men, but there was no correlation in women (50). In this study, we found that the abundance of *Bacteroides* of male mice in the HFD-fed group was reduced more than 2.5 times, and a high dose of HON significantly increased their abundance of it and the concentration of cecal SCFAs, but there was no significant change in female mice. Correlation analysis also showed that the abundance of *Bacteroides* was positively correlated with the gut acetate levels in male mice, but there was no such correlation in female mice, which was consistent with the abovementioned results. Moreover, SCFAs can effectively prevent obesity, dyslipidemia, and inflammation (51). Therefore, we speculate that HON may prevent obesity and related complications by producing SCFAs through posterior intestinal fermentation, and that sex is an important factor for different outcomes.

*Oscillospira* was negatively correlated with emaciation or lower BMI in children and adults (52, 53). A Western diet was associated with higher abundance of *Oscillospira* than a Mediterranean diet (48). A study by Amandine et al. showed that *Oscillospira's* abundance was positively correlated with obesity index and epididymal adipose tissue (54). A similar result was also found in this study, suggesting that HON may produce anti-obesity effects in HFD-fed mice by reversing the abundance of *Oscillospira*. In addition, the HFD-fed group of female mice did not have an increased abundance of *Oscillospira* compared with male mice. Studies also found that *Oscillospira*'s abundance was closely related to sex (42) and that there were sex-based differences in the changes in abundance caused by HFD, which, like *Akkermansia*, might be related to bile acid and sex hormone secretion (43). Correlation analysis showed that there was a significant positive correlation between *Oscillospira* and LBP, and TNF-α. These findings indicate that *Oscillospira* may facilitate the occurrence and development of obesity and related complications, and there is a large relationship between gut microbiota and sex.

LBP is an acute phase protein that specifically binds to LPS, promoting innate immunity and thus acts as an ideal biomarker of gut microbiota antigen inflammation. Gut microbiota can reduce serum LPS level (15), and its imbalance can lead to more bacterial lipopolysaccharides entering the bloodstream from the intestinal tract, resulting in inflammatory obesity and insulin resistance in obese mice (6, 48). In the present study, the levels of LBP in the high-fat group were significantly higher than those in the ND group, and was significantly reduced by adding HON. Correlation analysis showed that there was a significant positive correlation between *Dehalobacterium, Oscillospira, Muribaculaceae, rc4-4*, and *Oscillospira* and LBP, which was reduced by supplementation with HON compared to the HFD-fed group of mice. This indicates that HON may prevent the occurrence and development of obesity and obesity-related complications by decreasing the amount of LPS produced by gut microbiota.

Surprisingly, in this study, the H800 group had a reduced total food intake and there was no improvement insulin resistance in male mice in this group. We speculated that HON increased the concentration of SCFAs in the cecum, which were absorbed into the hepatic vein system through the intestines, promoted liver metabolism processes, such as gluconeogenesis, provided energy for liver metabolism (55). Elegant rodent studies have demonstrated that SCFAs have a suppressive effect on appetite and energy intake via central nervous system-related mechanisms and the gut–brain axis (56, 57). In addition, HON may have dose-dependent effects on gut microbiota. This study found that compared with other doses, the high dose of HON significantly reduced the richness of the gut microbiota, which also reduced the abundance of *Unclassified_Clostridiales* significantly in male mice, and reduced the abundance of *Muribaculaceae* more significantly in female mice.

## Conclusions

HON can prevent diet-induced obesity and associated complications by regulating gut microbiota and metabolites. Sex and dose may be important factors that deserve our attention while using this compound. This study provides a new perspective on the anti-obesity effect of the Chinese medicinal herbal extract HON, but its precise mechanism of action needs to be further verified.

## Data Availability Statement

The datasets generated for this study can be found in the NCBI - SRR10432877.

## Ethics Statement

The animal study was reviewed and approved by Ethics of Animal Experiments of Hunan Agriculture University.

## Author Contributions

YD, ZF, XH, and ZS designed the experiment and revised the manuscript. YD, HL, LC, and TP carried out the animal trials and sample analysis. YD and XG did some data analysis work and editor of the paper. ZF, XH, and ZS is responsible for the integrity of the work as a whole. All authors reviewed and approved the final manuscript.

### Conflict of Interest

The authors declare that the research was conducted in the absence of any commercial or financial relationships that could be construed as a potential conflict of interest.
