# Supplementary material for: Honokiol Ameliorates High-Fat-Diet-Induced Obesity of Different Sexes of Mice by Modulating the Composition of the Gut Microbiota
Source: Front Immunol. 2019 Dec 11;10:2800. doi: 10.3389/fimmu.2019.02800 (PMC6917612; doi:10.3389/fimmu.2019.02800)
Supplement: Supplementary file 1 [file Table_1.DOC]

Table S1. HON supplements alter the diversity of gut microbiota in HFD-fed female mice

| Parameters | | ND | HFD | H200 | H400 | H800 | P |
| --- | --- | --- | --- | --- | --- | --- | --- |
| OTUs | | 1308.80±74.04^a^ | 1247.20±116.42^a^ | 1322.80±187.05^a^ | 1136.00±153.72^a^ | 779.40±260.46^b^ | ＜0.01 |
| Richness | Chao1 | 1361.65±143.064^a^ | 1297.93±152.76^a^ | 1419.33±221.61^a^ | 1180.78±165.11^a^ | 861.10±273.13^b^ | ＜0.01 |
|  | ACE | 1384.60±165.88^a^ | 1300.91±155.49^a^ | 1433.79±212.81^a^ | 1203.32±177.18^a^ | 883.09±289.56^b^ | ＜0.01 |
| Diversity | Shannon | 7.21±0.23^a^ | 6.26±0.68^ab^ | 6.61±1.01^a^ | 5.47±0.80^b^ | 4.91±0.99^b^ | ＜0.01 |
|  | Simpson | 0.97±0.01^a^ | 0.91±0.06^ab^ | 0.93±0.05^a^ | 0.84±0.07^b^ | 0.85±0.07^b^ | ＜0.01 |

Data are expressed as means ± SE (n = 5). ND: nomal chow diet, HFD: high fat diet, HON: honokiol. H200, H400 and H800 correspond to honokiol supplemented with 200, 400 and 800mg/kg in high fat diet, respectively. Mean values with different letters indicate statistical significance (*p* < 0.05).

Table S2. HON supplements alter the composition of gut microbiota in HFD-fed female mice at genus level.

| Parameters | ND | HFD | H200 | H400 | H800 | P |
| --- | --- | --- | --- | --- | --- | --- |
| *Akkermansia* | 4.72±8.06^c^ | 27.06±10.53^ab^ | 17.92±15.36b^c^ | 37.60±11.34^a^ | 32.78±15.19^ab^ | ＜0.01 |
| *Unclassified_*  *Clostridiales* | 28.72±6.39^ab^ | 18.60±7.59^ab^ | 30.56±11.60^a^ | 19.52±7.99^ab^ | 17.12±10.04^b^ | 0.08 |
| *Muribaculaceae* | 30.34±6.14^a^ | 13.92±1.51^b^ | 6.20±3.37^c^ | 4.42±2.46^cd^ | 0.00±0.00^d^ | ＜0.01 |
| *Unclassified_*  *Ruminococcaceae* | 6.40±0.30^b^ | 6.24±2.75^b^ | 12.14±3.68^a^ | 5.62±1.01^b^ | 6.08±5.46^b^ | ＜0.05 |
| *ParaBacteroides* | 4.20±2.12 | 6.94±2.80 | 7.32±5.56 | 8.70±9.36 | 8.60±9.25 | 0.818 |
| *Unclassified_*  *Lachnospiraceae* | 4.88±1.75 | 3.50±1.38 | 5.92±3.30 | 4.86±0.86 | 6.02±4.30 | 0.574 |
| *Oscillospira* | 6.18±1.11^a^ | 5.96±1.77^a^ | 3.34±2.19^b^ | 3.14±1.15^b^ | 1.98±1.16^b^ | ＜0.01 |
| *[Ruminococcus]* | 1.74±0.51 | 2.94±0.65 | 2.56±0.86 | 1.92±0.85 | 3.08±2.20 | 0.311 |
| *rc4-4* | 2.86±0.90^a^ | 3.38±1.28^a^ | 2.14±1.49^ab^ | 1.62±2.17^ab^ | 0.54±1.10^b^ | ＜0.05 |
| *Bacteroides* | 1.22±0.68 | 2.38±2.24 | 1.28±1.22 | 3.70±4.41 | 0.78±0.95 | 0.313 |
| *Roseburia* | 0.02±0.04 | 0.04±0.05 | 2.14±1.65 | 2.42±0.83 | 3.84±7.60 | 0.380 |
| *Lactobacillus* | 1.22±0.77 | 1.68±1.45 | 1.06±1.44 | 0.70±0.68 | 0.50±0.51 | 0.447 |
| *Lactococcus* | 0.36±0.27 | 1.42±1.30 | 1.40±1.18 | 0.70±0.57 | 0.40±0.24 | 0.146 |
| *Phascolarctobacterium* | 0.00±0.00 | 0.00±0.00 | 0.00±0.00 | 0.00±0.00 | 4.28±7.54 | 0.210 |
| *Dorea* | 0.32±0.22 | 1.22±1.11 | 1.38±1.32 | 0.76±0.73 | 0.48±0.13 | 0.249 |
| *Unclassified_*  *Enterobacteriaceae* | 0.00±0.00^b^ | 0.32±0.49^b^ | 0.04±0.05^b^ | 0.60±0.81^b^ | 2.64±2.07^a^ | ＜0.01 |
| *Ruminococcus* | 1.02±0.37^a^ | 0.78±0.66^ab^ | 0.56±0.30^ab^ | 0.28±0.16^b^ | 0.26±0.24^b^ | ＜0.05 |
| *Adlercreutzia* | 0.46±0.25 | 0.20±0.16 | 0.86±0.72 | 0.68±0.57 | 0.60±0.57 | 0.335 |
| *Coprococcus* | 0.36±0.11 | 0.26±0.05 | 0.66±0.32 | 0.66±0.46 | 0.36±0.36 | 0.149 |
| *Unclassified_*  *Erysipelotrichaceae* | 0.08±0.11^b^ | 0.50±0.20^a^ | 0.56±0.48^a^ | 0.58±0.13^a^ | 0.58±0.28^a^ | ＜0.05 |
| *Unclassified_*  *Peptostreptococcaceae* | 1.16±0.71^a^ | 0.38±0.30^b^ | 0.22±0.39^b^ | 0.46±0.63^b^ | 0.00±0.00^b^ | ＜0.05 |
| *Bifidobacterium* | 1.30±0.78^a^ | 0.00±0.00^b^ | 0.00±0.00^b^ | 0.00±0.00^b^ | 0.00±0.00^b^ | ＜0.01 |
| *Dehalobacterium* | 0.34±0.17^a^ | 0.32±0.16^a^ | 0.28±0.13^a^ | 0.08±0.11^b^ | 0.00±0.00^b^ | ＜0.01 |
| *Mucispirillum* | 0.32±0.11 | 0.20±0.28 | 0.26±0.32 | 0.14±0.19 | 0.12±0.08 | 0.588 |

Data are expressed as means ± SE (n = 5). ND: nomal chow diet, HFD: high fat diet, HON: honokiol. H200, H400 and H800 correspond to honokiol supplemented with 200, 400 and 800mg/kg in high fat diet, respectively. Mean values with different letters indicate statistical significance (*p* < 0.05).

| Parameters | | ND | HFD | H200 | H400 | H800 | P |
| --- | --- | --- | --- | --- | --- | --- | --- |
| OTUs | | 1254.40+233.23^AB^ | 1469.60+102.28^A^ | 1152.00+130.22^B^ | 1180.00+96.21^B^ | 659.20+341.98^C^ | ＜0.01 |
| Richness | Chao1 | 1338.67+228.25^A^ | 1560.26+181.60^A^ | 1250.84+149.29^A^ | 1346.54+200.19^A^ | 748.60+385.22^B^ | 0.01 |
|  | ACE | 1351.20+229.69^A^ | 1583.19+157.91^A^ | 1306.71+154.27^A^ | 1352.86+183.53^A^ | 758.07+416.10^B^ | 0.01 |
| Diversity | Shannon | 6.78+0.89^A^ | 7.13+0.25^A^ | 5.40+0.51^B^ | 5.37+0.78^B^ | 4.12+0.53^C^ | ＜0.01 |
|  | Simpson | 0.94+0.031^A^ | 0.96+0.0095^A^ | 0.86+0.037^B^ | 0.85+0.072^B^ | 0.78+0.033^C^ | ＜0.01 |

Table S3. HON supplements alter the diversity of gut microbiota in HFD-fed male mice.

Data are expressed as means ± SE (n = 5). ND: nomal chow diet, HFD: high fat diet, HON: honokiol. H200, H400 and H800 correspond to honokiol supplemented with 200, 400 and 800mg/kg in high fat diet, respectively. Mean values with different letters indicate statistical significance (*p* < 0.05).

Table S4. HON supplements alter the composition of gut microbiota in HFD-fed male mice at genus level.

| Parameters | ND | HFD | H200 | H400 | H800 | P |
| --- | --- | --- | --- | --- | --- | --- |
| *Akkermansia* | 14.20±11.40^B^ | 6.62±6.46^B^ | 34.22±6.70^A^ | 29.36±16.40^A^ | 43.44±6.23^A^ | ＜0.01 |
| *Unclassified_*  *Clostridiales* | 28.88±9.56^A^ | 26.32±6.01^AB^ | 17.82±2.87^B^ | 21.40±7.47^AB^ | 4.94±5.58^C^ | ＜0.01 |
| *Parabacteroides* | 4.94±3.20^B^ | 13.42±5.27^AB^ | 9.90±7.07^B^ | 20.36±7.62^A^ | 7.66±5.95^B^ | ＜0.01 |
| *Muribaculaceae* | 23.26±8.45^A^ | 15.40±2.32^B^ | 5.30±2.07^C^ | 0.00±0.00^C^ | 0.00±0.00^C^ | ＜0.01 |
| *Unclassified_*  *Ruminococcaceae* | 5.98±1.69^AB^ | 8.80±3.46^A^ | 6.96±2.50^AB^ | 4.32±0.81^B^ | 4.04±2.75^B^ | ＜0.05 |
| *Oscillospira* | 5.22±2.17^B^ | 10.28±6.92^A^ | 3.50±2.16^B^ | 3.10±1.38^B^ | 1.40±1.25^B^ | ＜0.01 |
| *Unclassified_*  *Lachnospiraceae* | 2.60±0.40 | 4.30±1.43 | 4.84±4.34 | 4.82±1.02 | 5.28±5.52 | 0.721 |
| *Bacteroides* | 3.94±3.78^AB^ | 1.48±1.00^B^ | 1.20±1.61^B^ | 0.86±0.64^B^ | 6.72±6.81^A^ | 0.085 |
| *rc4-4* | 2.98±0.96^AB^ | 3.34±1.57^AB^ | 4.28±2.71^A^ | 3.00±3.75^AB^ | 0.60±1.34^B^ | 0.182 |
| *[Ruminococcus]* | 1.00±0.41 | 2.72±3.22 | 2.84±1.41 | 2.14±1.02 | 1.26±0.90 | 0.337 |
| *Fusobacterium* | 0.00±0.00^B^ | 0.00±0.00^B^ | 0.00±0.00^B^ | 0.00±0.00^B^ | 8.48±12.04^A^ | 0.77 |
| *Roseburia* | 0.02±0.04^B^ | 0.12±0.13^B^ | 1.64±1.03^AB^ | 2.96±2.86^A^ | 1.98±1.72^AB^ | ＜0.05 |
| *Bilophila* | 0.00±0.00^B^ | 0.00±0.00^B^ | 0.00±0.00^B^ | 1.72±3.85^AB^ | 3.80±3.78^A^ | 0.082 |
| *Dorea* | 0.20±0.16 | 0.58±0.74 | 2.18±2.40 | 0.50±0.29 | 1.62±2.16 | 0.213 |
| *Phascolarctobacterium* | 0.00±0.00 | 0.00±0.00 | 0.00±0.00 | 1.30±2.91 | 2.72±3.73 | 0.205 |
| *Unclassified_*  *Enterobacteriaceae* | 0.00±0.00^B^ | 0.06±0.05^B^ | 0.12±0.22^B^ | 0.76±0.71^B^ | 2.96±2.57^A^ | ＜0.01 |
| *Ruminococcus* | 1.12±0.40^AB^ | 1.40±0.62^A^ | 0.72±0.38^B^ | 0.22±0.04^C^ | 0.06±0.05^C^ | ＜0.01 |
| *Unclassified_*  *Erysipelotrichaceae* | 0.18±0.16 | 0.48±0.47 | 0.62±0.52 | 0.40±0.20 | 0.80±0.98 | 0.487 |
| *Lactobacillus* | 0.96±0.60^B^ | 0.38±0.08^B^ | 0.34±0.28^B^ | 0.28±0.27^B^ | 0.10±0.00^A^ | ＜0.01 |
| *Allobaculum* | 0.52±0.55 | 0.18±0.40 | 0.32±0.15 | 0.44±0.87 | 0.30±0.40 | 0.870 |
| *Adlercreutzia* | 0.48±0.26^AB^ | 0.24±0.11^BC^ | 0.60±0.32^A^ | 0.24±0.15^BC^ | 0.16±0.11^C^ | ＜0.05 |
| *Coprococcus* | 0.26±0.11^B^ | 0.28±0.11^B^ | 0.30±0.19^B^ | 0.60±0.27^A^ | 0.30±0.24^B^ | 0.069 |
| *Lactococcus* | 0.06±0.05^B^ | 0.70±0.63^A^ | 0.36±0.27^AB^ | 0.24±0.15^B^ | 0.12±0.08^B^ | ＜0.05 |
| *Mucispirillum* | 0.14±0.17 | 0.58±0.62 | 0.16±0.11 | 0.38±0.24 | 0.16±0.21 | 0.174 |
| *Unclassified_*  *Peptostreptococcaceae* | 0.52±0.37 | 0.40±0.26 | 0.46±0.68 | 0.02±0.04 | 0.00±0.00 | 0.093 |
| *Dehalobacterium* | 0.30±0.12^A^ | 0.46±0.19^A^ | 0.30±0.12^A^ | 0.00±0.00^B^ | 0.00±0.00^B^ | ＜0.01 |
| *Blautia* | 0.18±0.22^AB^ | 0.28±0.18^A^ | 0.08±0.13^AB^ | 0.00±0.00^B^ | 0.10±0.17^AB^ | 0.100 |
| *Unclassified_*  *[Mogibacteriaceae]* | 0.12±0.04 | 0.08±0.04 | 0.14±0.05 | 0.16±0.05 | 0.14±0.15 | 0.603 |
| *Unclassified_*  *Christensenellaceae* | 0.14±0.05 | 0.12±0.04 | 0.16±0.13 | 0.10±0.00 | 0.08±0.08 | 0.520 |
| *Bifidobacterium* | 0.56±0.39^A^ | 0.00±0.00^B^ | 0.00±0.00^B^ | 0.00±0.00^B^ | 0.00±0.00^B^ | ＜0.01 |
| *Acinetobacter* | 0.14±0.15 | 0.16±0.25 | 0.04±0.05 | 0.08±0.08 | 0.02±0.04 | 0.457 |

Data are expressed as means ± SE (n = 5). ND: nomal chow diet, HFD: high fat diet, HON: honokiol. H200, H400 and H800 correspond to honokiol supplemented with 200, 400 and 800mg/kg in high fat diet, respectively.Mean values with different letters indicate statistical significance (*p* < 0.05).
